# Serum Sphingosine-1-phosphate level and peritonitis in peritoneal dialysis patients

**DOI:** 10.1080/0886022X.2020.1805763

**Published:** 2020-08-13

**Authors:** Qiong Bai, Hong-Xia Guo, Chun-Yan Su, Qing-Feng Han, Tao Wang, Wen Tang

**Affiliations:** Department of Nephrology, Peking University Third Hospital, Beijing, China

**Keywords:** Sphingosine-1-phosphate, peritonitis, intestinal barrier, peritoneal dialysis

## Abstract

**Background:**

Given the important role of Sphingosine-1-phosphate (S1P) in maintaining the hemostasis in intestinal barrier function and regulation of inflammation and immune, we hypothesize that S1P might be a biomarker to predict peritonitis in peritoneal dialysis (PD) patients.

**Methods:**

In this case-control study, 78 stable, continuous ambulatory peritoneal dialysis patients were enrolled and followed for the episode of PD associated peritonitis. Patients were divided into two groups by whether or not they had peritonitis during follow-up: non-peritonitis (*n* = 65) and peritonitis (*n* = 13) group. S1P was analyzed by enzyme-linked immunosorbent assay. Logistic regression analysis was used to assess factors associated with peritonitis. The variables identified by univariable regression models (*p* < 0.1) were further selected into the multivariable logistic regression model to determine whether they could independently affect peritonitis.

**Results:**

Patients with peritonitis had a lower level of S1P than that of patients without peritonitis (1.3 ng/mL IQ 0.8, 3.6 ng/mL vs. 2.8 ng/mL IQ 1.5, 5.4 ng/mL, *p* = 0.018). The peritonitis group had lower serum albumin, lower blood leukocyte, lower hemoglobin and lower platelet count as compared to the non-peritonitis group. Logistic regression analysis showed that S1P (OR = 0.381, 95% CI = 0.171–0.848, *p* = 0.018), blood leukocyte count (OR = 0.438, 95% CI = 0.207–0.925, *p* = 0.030), and serum albumin (OR = 0.732, 95% CI = 0.556–0.962, *p* = 0.025) were independent factors associated with peritonitis in the present PD population.

**Conclusion:**

Our study showed that S1P was an independent determinant of subsequent peritonitis in PD patients. S1P might serve as a biomarker to predict peritonitis in PD patients.

## Introduction

Peritoneal dialysis (PD) is currently a renal replacement therapy (RRT) modality for the treatment of end-stage renal disease (ESRD) [1]. However, PD related peritonitis is a serious complication which is the greatest contributor to infection-related morbidity, including risk for hospitalization, and temporary or permanent transfer to hemodialysis [2–4]. Risk factors for PD related peritonitis have been well documented, with bacterial translocation from the gut well accepted as a potential cause of peritonitis [5]. In patients with liver cirrhosis, spontaneous bacterial peritonitis resulting from abnormal intestinal permeability and bacterial overgrowth have also been well documented as risk factors [6,7]. In addition for patients with renal failure, impaired gastrointestinal barrier function and increased gut permeability promote the translocation of bacteria [8–14].

In the human gut, the integrity of the epithelial cell layer, which forms an important barrier for gastrointestinal tract, strongly depends on specialized structures involved in cell-cell contacts, including tight junctions, adherent junctions, and desmosomes [15]. Sphingosine-1-phosphate (S1P) has recently shown to be a potent bioactive sphingolipid metabolite that regulates diverse cellular processes, which are important for inflammation, immune responses and barrier regulation [16–22]. Previous studies have reported that S1P protects intestinal epithelial cell from apoptosis, maintains cell-cell contacts in gut epithelial cells and ultimately plays a critical role in modulating the integrity of the intestinal barrier [23].

Given the important role of S1P in maintaining hemostasis in gut barrier function and achieving immune regulation, we hypothesize that S1P might be a biomarker to predict peritonitis in PD patients. Therefore, in the present study, we investigated the association of serum S1P level and risk of peritonitis in PD patients.

## Methods

In this case-control study, patients who visited our PD clinic for routine clinic evaluation from 19 December 2016 to 20 February 2017 and had their blood tested were grouped according to whether or not they had peritonitis by the follow-up date of 31 January 2018. Seventy-eight stable, continuous ambulatory peritoneal dialysis (CAPD) patients were enrolled from our PD program. All patients received CAPD treatment with dialysate containing 132 mmol/L of sodium and 1.5 or 2.5% of glucose concentration (Dianeal, Baxter Healthcare, Guangzhou, China). In our center, dialysis policy was implemented as previously described. Gradual increases in the PD dose were prescribed while the residual kidney function no longer could maintain the fluid balance and the total Kt/V reached 1.7 if possible. Consequently, all patients received 3–5 units of exchange (2 L per exchange) per day during the follow-up.

Exclusion criteria were (1) the duration of patients treated with CAPD was less than 3 months before the inclusion period; (2) patients were age <18 years; (3) patients with overt heart failure (NYHA grade III–IV); (4) patients had autoimmune disease; (5) patients had history of malignant disease; (6) patients had history of liver cirrhosis; (7) patients had acute or chronic intestinal inflammatory disease. The study was approved by the Medical Ethical Committee of Peking University.

Clinical data, demographic data and laboratory examinations were prospectively recorded in an electronic database and included age, gender, dialysis vintage, cause of primary renal disease, diabetes mellitus status, body mass index (BMI), dates and microbiology of peritonitis episodes, and patient baseline assay results.

Peritonitis was diagnosed when at least two of the following are present: (1) clinical features consistent with peritonitis, i.e., abdominal pain and/or cloudy dialysis effluent; (2) dialysis effluent white cell count >100/μL or >0.1 × 10^9^/L (after a dwell time of at least 2 h), with >50% polymorphonuclear; and (3) positive dialysis effluent culture.

### Measurement of biochemical parameters

Serum albumin was determined by the bromcresol green method. Other biochemical indices, such as blood urea, serum creatinine, uric acid, serum potassium, total cholesterol, triglyceride, ferritin, high-sensitivity C-reactive protein (hs-CRP) and parathyroid hormone (PTH), were determined using standard methods.

### Measurement of serum S1P

S1P was analyzed by enzyme-linked immunosorbent assay (ELISA) using a commercial kit (Nordic BioSite AB, Sweden). The assay was conducted according to the manufacturer’s instructions. A 96-well plate was coated with S1P in advance and blocked to reduce nonspecific binding. After mixing the S1P standards and serum samples with the anti-S1P antibody, we added the mixture to the plate. The antibody competes for binding to S1P bound to the plate or in the serum sample. Following a 1-h incubation period and five washes of the plate, we added streptavidin–horseradish peroxidase (HRP) to the plate. After an additional incubation period, we added 3,30,5,50-tetramethylbenzidine (TMB) substrate. We stopped the reaction by adding sulfuric acid. We then measured the absorbance at 450 nm within 15 min. The concentration of S1P in the serum samples was determined by comparing values to those in a standard curve.

### Measurement of dialysis adequacy and residual renal function

Residual renal function was measured as glomerular filtration rate (GFR) using the mean rate for urea and creatinine clearance [24]. The dialysis dose was measured according to the instilled dialysate volume. Small solute removal was determined by measurement of total (peritoneal dialysis and renal) weekly urea Kt/V using standard methods [25]. The contributions to total Kt/V (tKt/V) by peritoneal dialysis (pKt/V) and residual renal function (rKt/V) were estimated separately. The volume of urea distribution (V) was derived using Watson’s formula [26].

### Statistical analysis

Continuous variables are expressed as means ± standard deviation for continuous normally distributed variables, and medians [interquartile range; IQR] for continuous variables that were not normally distributed; categorical variables are expressed as percentages. Peritonitis and non-peritonitis groups were compared using an independent t-test or Mann-Whitney's test, depending on the data distribution. For comparison of categorical variables in two groups, such as the proportion of diabetics or gender distribution, chi-square tests were employed. Logistic regression analysis was used to assess factors associated with peritonitis. The variables identified by univariable regression models (*p* < 0.1) were further selected into the multivariable logistic regression model to determine whether they could independently affect peritonitis. Multivariable logistic regression analysis was used with the entry method. Results of the regression models are reported as odds ratio (OR) with 95% confidence interval (95% CI). All tests were two sided and *p* < 0.05 was considered to be statistically significant. Statistical analysis was performed using SPSS software, version 25.0 (SPSS Inc., Chicago, Illinois, USA).

## Results

### Demographic characteristics and clinical profile of the study population

**Figure 1.**
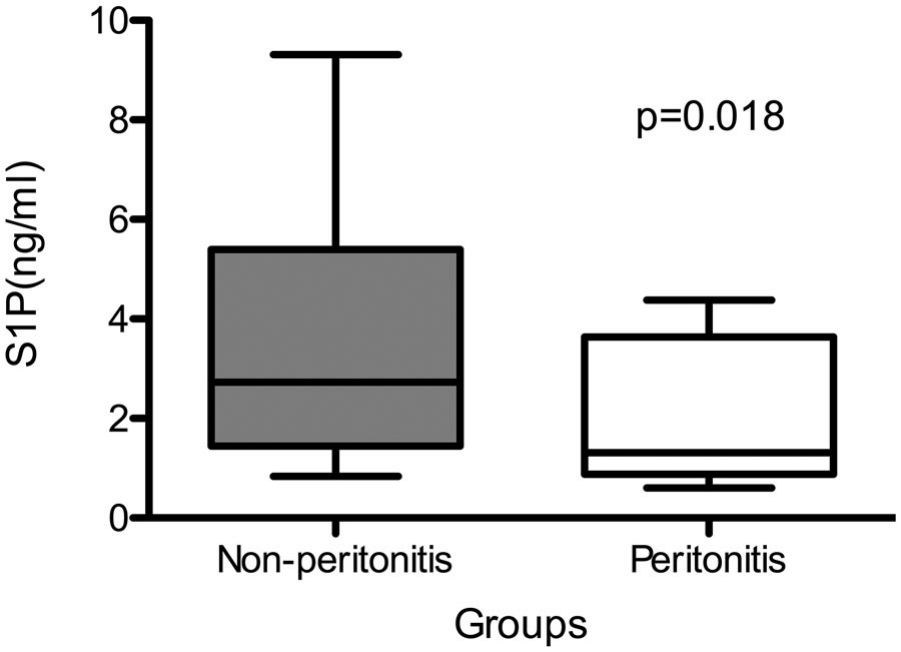
Sphingosine 1-phosphate level in patients with or without peritonitis during follow-up.

A total of 78 PD patients were included. During the follow-up, a total of 13 episodes of peritonitis were recorded. Gram negative, Gram positive and culture negative peritonitis occurred in 5, 3 and 5 patients, respectively. The culture of patients with Gram negative bacilli was all *Escherichia coli*. The culture of patients with Gram positive cocci were all coagulase-negative staphylococci. All the patients’ treatment protocol followed the 2016 ISPD guideline, with initial treatment of vancomycin and ceftazidime in our center. Patients were divided into two groups according to whether or not they had peritonitis during follow-up, resulting in a non-peritonitis (*n* = 65) and peritonitis (*n* = 13) group. The baseline comparisons between the two groups are described in Table 1. The time interval between laboratory tests and subsequent peritonitis was 10.2 (IQ 2.26, 10.62) months. Patients with peritonitis had a lower level of S1P than that of patients without peritonitis (1.3 ng/mL IQ 0.8, 3.6 ng/mL vs. 2.8 ng/mL IQ1.5, 5.4 ng/mL, *p* = 0.018) (Figure 1). In addition, the peritonitis group had lower serum albumin (34.4 ± 4.7 g/L vs. 37.0 ± 3.0 g/L, *p* = 0.010), lower blood leukocyte (6.2 ± 1.2 × 10^9^/L vs. 7.7 ± 2.0 × 10^9^/L, *p* = 0.007), lower hemoglobin (106.7 ± 9.3 g/L vs. 115.1 ± 11.0 g/L, *p* = 0.012) and lower platelet count (141.8 ± 63.3 × 10^12^/L vs. 204.8 ± 67.4 × 10^12^/L, *p* = 0.003) as compared to the non-peritonitis group. There was no significant difference between the peritonitis and non-peritonitis patients with respect to the other variables.

**Table 1.** Clinical parameters at baseline for groups of peritonitis and non-peritonitis patients.

Variable	Non-peritonitis *n* = 65	Peritonitis *n* = 13	*p*-Value
Gender (male)	50.8%	53.8%	1.000
Age (years)	64.3 ± 11.6	65.3 ± 11.4	0.780
Diabetes mellitus (%)	66.1%	69.2%	1.000
BMI (kg/m^2^)	22.7 ± 3.7	23.6 ± 3.6	0.977
Dialysis vintage (years)	5.2 ± 3.5	3.8 ± 2.9	0.176
Previous peritonitis episode (%)	29.2%	30.8%	0.912
Residual GFR (ml/min/1.73 m^2^)	0 (0, 12.0)	0 (0, 7.9)	0.280
Peritoneal Ccr (ml/min)	42.2 ± 9.3	41.4 ± 9.5	0.777
Residual Ccr (ml/min)	0 (0, 14.9)	0 (0, 16.9)	0.477
Total Ccr (ml/min)	51.6 ± 14.2	48.2 ± 9.0	0.409
Peritoneal Kt/V	1.7 ± 0.3	1.6 ± 0.4	0.531
Residual renal Kt/V	0 (0, 0.2)	0 (0, 0.4)	0.685
Total Kt/V	1.9 ± 0.4	1.8 ± 0.3	0.524
Instilled dialysate volume (L/day)	8.6 ± 1.3	8.3 ± 1.5	0.398
Serum S1P (ng/ml)	2.8 (1.5, 5.4)	1.3 (0.8, 3.6)	0.018
Creatinine (µmol/L)	874 (763.5, 1012)	775 (696.5, 1086.5)	0.503
Urea (mmol/L)	17.7 ± 4.6	17.5 ± 3.3	0.488
Uric acid (µmol/L)	383 (340.5, 445)	394 (364.5, 429.7)	0.673
Albumin (g/L)	37.0 ± 3.0	34.4 ± 4.7	0.010
Leukocyte (×10^9^/L)	7.7 ± 2.0	6.2 ± 1.2	0.007
Hemoglobin (g/L)	115.1 ± 11.0	106.7 ± 9.3	0.012
Platelet (×10^12^/L)	204.8 ± 67.4	141.8 ± 63.3	0.003
Serum potassium (mmol/L)	4.3 ± 0.6	4.3 ± 0.4	0.874
Total cholesterol (mmol/L)	4.9 ± 1.1	4.3 ± 1.0	0.093
Triglyceride (mmol/L)	2.7 ± 1.9	1.7 ± 1.3	0.098
PTH (pg/mL)	163.8 (65.1, 352.2)	147.1 (102.5, 253.6)	0.945
Ferritin (ng/mL)	264 (187, 343)	248.6 (173, 361)	0.536
hs-CRP (mg/L)	4.9 (1.6, 17.2)	3.1 (0.8, 7.7)	0.230

BMI: body mass index; Residual GFR: residual glomerular filtration rate; Ccr: creatinine clearance rate; S1P: sphingosine 1-phosphate; PTH: parathyroid hormone; hs-CRP: high-sensitivity C-reactive protein.

### Logistic regression analysis

Logistic regression analysis was performed to identify independent factors associated with peritonitis. The results of the logistic regression analysis are shown in Table 2. The following variables reached statistical significance at a p-value of 0.1 or lower for the univariable logistic regression: S1P (OR = 0.652, 95% CI = 0.427–0.997, *p* = 0.048), leukocyte count (OR = 0.554, 95% CI = 0.348–0.882, *p* = 0.013), hemoglobin (OR = 0.928, 95% CI = 0.872–0.987, *p* = 0.017), platelet (OR = 0.982, 95% CI = 0.970–0.994, *p* = 0.004), serum albumin (OR = 0.806, 95% CI = 0.673–0.965, *p* = 0.019) and total cholesterol (OR = 0.595, 95% CI = 0.322–1.101, *p* = 0.098). The results of the multivariable logistic regression model showed that S1P (OR = 0.381, 95% CI = 0.171–0.848, *p* = 0.018), blood leukocyte count (OR = 0.438, 95% CI = 0.207–0.925, *p* = 0.030), and serum albumin (OR = 0.732, 95% CI = 0.556–0.962, *p* = 0.025) were independent factors associated with episode of peritonitis in the PD study population.

**Table 2.** Logistic regression analysis of the independent factors associated with peritonitis in PD patients.

	Univariate logistic regression			Multivariate logistic regression		
Variable	OR	95% CI	*p*-Value	OR	95% CI	*p*-Value
Gender (male)	1.131	0.343–3.733	0.839			
Age (years)	1.008	0.956–1.063	0.777			
DM	0.868	0.239–3.152	0.829			
BMI (kg/m^2^)	1.002	0.859–1.169	0.976			
Dialysis vintage (years)	0.873	0.716–1.064	0.179			
Previous peritonitis episode	1.076	0.295–3.922	0.912			
Residual GFR (ml/min/1.73 m^2^)	0.951	0.872–1.038	0.264			
Peritoneal Ccr (ml/min)	0.991	0.929–1.056	0.774			
Residual Ccr (ml/min)	0.987	0.942–1.034	0.578			
Total Ccr (ml/min)	0.978	0.929–1.030	0.403			
Peritoneal Kt/V	0.556	0.091–3.407	0.526			
Residual Kt/V	0.867	0.155–4.837	0.870			
Total Kt/V	0.873	0.716–1.064	0.179			
Instilled dialysis volume (L/day)	1.000	0.999–1.000	0.393			
Serum S1P (ng/ml)	0.652	0.427–0.997	0.048	0.381	0.171 − 0.848	0.018
Creatinine (µmol/L)	0.999	0.997–1.002	0.618			
Urea (mmol/L)	0.987	0.861–1.133	0.857			
Uric acid (µmol/L)	1.000	0.991–1.008	0.934			
Albumin (g/L)	0.806	0.673–0.965	0.019	0.732	0.556–0.962	0.025
Leukocyte (×10^9^/L)	0.554	0.348–0.882	0.013	0.438	0.207–0.925	0.030
Hemoglobin (g/L)	0.928	0.872–0.987	0.017	0.924	0.845–1.010	0.082
Platelet (×10^12^/L)	0.982	0.970–0.994	0.004	1.002	0.984–1.019	0.861
Serum potassium (mmol/L)	0.921	0.339–2.502	0.872			
Total cholesterol (mmol/L)	0.595	0.322–1.101	0.098	0.876	0.387–1.985	0.752
Triglyceride (mmol/L)	0.563	0.283–1.121	0.102			
PTH (pg/mL)	1.000	0.998–1.002	0.786			
Ferritin (ng/mL)	0.998	0.994–1.002	0.395			
hs-CRP (mg/L)	0.952	0.880–1.030	0.218			
Constant						0.002

OR: Odds ratio; 95% CI: 95% confidence interval; DM: diabetes mellitus; BMI: body mass index; Residual GFR: residual glomerular filtration rate; Ccr: creatinine clearance rate; S1P: sphingosine 1-phosphate; PTH: parathyroid hormone; hs-CRP: high-sensitivity C-reactive protein.

## Discussion

In the present study, we found that patients with subsequent PD related peritonitis had significantly lower serum S1P levels than patients without peritonitis. Most importantly, the serum S1P level was a significant factor associated with peritonitis after adjusting for other confounding factors.

Peritonitis is still the major cause of patient technique failure and dropout from PD therapy. A number of established risk factors have been discovered to be associated with PD related peritonitis. Recently, much attention has been paid to the leak gut hypothesis [27]. Gastrointestinal problems, such as constipation and enteritis, have been reported to be associated with peritonitis due to enteric organisms in PD patients [28–30]. Our previous study has proved that gastrointestinal symptoms predict peritonitis rates in PD patients [30].

The mechanisms by which micro-organisms from the intestinal lumen gain access to the peritoneal cavity and cause enteric peritonitis may depend on the integrity of the intestinal wall, bacterial overgrowth status in the intestinal lumen and host peritoneal defense mechanisms [31,32]. Several observations have provided indirect evidence of intestinal barrier dysfunction in patients and animals with advanced chronic kidney disease [13]. Damage to the intestinal barrier and increased intestinal permeability may precede and promote translocation of bacteria and endotoxins [14,33]. Intestinal bacterial translocation in both chronic renal failure patients and experimental chronic renal failure models have been reported [34–36]. Studies also have shown that colon wall inflammation, along with destruction of the intestinal epithelial tight junction barrier, could lead to translocation of bacterial DNA and lipopolysaccharide into the bloodstream [34,37].

S1P is a bioactive lysophospholipid with important biological functions, which include cell adhesion, barrier regulation, migration, immune cell trafficking, proliferation and survival [16–22]. Many studies have reported that S1P protects intestinal epithelial cells, enhances the intestinal barrier function and ensures its integrity from apoptosis, proliferation, and migration by regulating the expression of adherens junction proteins [23,38–41]. A recent study has also shown that S1P signaling affects the migration of peritoneal B Cell populations and influences the production of intestinal IgA, suggesting that S1P signaling may be a target for modulating B cell function in inflammatory intestinal pathologies [42]. Therefore, the decreased S1P levels found in the present study may be an indicator for impaired intestinal barrier function and integrity, which facilitate the translocation of bacteria.

On the other hand, we found that serum S1P level was negatively associated with high-sensitivity C-reactive protein (hs-CRP) level (*r* = 0.61, *p* < 0.001), which indicates that S1P may also be associated with systemic inflammation. A recent study displayed similar phenomena, showing that the serum S1P level dramatically decreased and was inversely associated with disease severity in patients with sepsis [43]. We speculated that the disruption of the intestinal barrier function and integrity by a decrease in S1P level not only promotes translocation of bacteria but also may increase the levels of endotoxins, uremic toxins and lipopolysaccharides, which can potentially contribute to local and systemic inflammation and malnutrition. As systemic inflammation and malnutrition are well-known risk factors for PD related peritonitis [44–46], these risk factors may have predisposed the patients in the present study with decreased S1P concentrations to peritonitis.

In the present study, baseline blood leukocyte count was found as a protective factor for the episode of peritonitis in the PD patients. As blood leukocyte level was considered as a general marker of immunocompetence [47], total blood leukocyte count might provide a representation of the immune response to peritonitis and play an important role in the inflammatory response in PD patients. However, components of leukocytes, such as neutrophils, lymphocytes and macrophages, which could represent the immunocompetence [48,49], were not collected in the study. Serum potassium level was not a risk factor for PD peritonitis, possibly because of the relatively small sample of only 8.9% patients who had hypokalemia.

Strengths of the present study included the availability of serum S1P in stable CAPD patients. To our knowledge, this was the first study to evaluate the association of serum S1P with subsequent peritonitis in PD patients.

There were some limitations in the present study. The major limitation of this study was its observational design. Therefore, the independent link between peritonitis and level of S1P cannot demonstrate cause and effect. Second, the time interval between S1P measurement and subsequent peritonitis was a relatively long time period. Third, the total number of CAPD patients and patients with peritonitis was relatively small. In addition, we did not collect data about using laxatives. Thus, it is not clear whether laxatives might contribute to the difference in S1P levels.

In conclusion, this study showed serum S1P was independently and negatively associated with peritonitis in PD patients. Serum S1P might serve as a biomarker to predict peritonitis in PD patients.
